# The regenerative effect of different growth factors and platelet lysate on meniscus cells and mesenchymal stromal cells and proof of concept with a functionalized meniscus implant

**DOI:** 10.1002/term.3218

**Published:** 2021-05-21

**Authors:** Michella H. Hagmeijer, Jasmijn V. Korpershoek, João F. Crispim, Li‐Ting Chen, Pascal Jonkheijm, Aaron J. Krych, Daniel B.F. Saris, Lucienne A. Vonk

**Affiliations:** ^1^ Department of Orthopedics University Medical Center Utrecht Utrecht The Netherlands; ^2^ Developmental Bioengineering University of Twente Enschede The Netherlands; ^3^ Department of Molecules and Materials MESA+ Institute for Nanotechnology University of Twente Enschede The Netherlands; ^4^ Department of Orthopedic Surgery and Sports Medicine Mayo Clinic Rochester Minnesota USA

**Keywords:** CMI®, growth factors, meniscus, mesenchymal stromal cells, migration, platelet‐rich plasma, proliferation, regeneration

## Abstract

Meniscus regeneration could be enhanced by targeting meniscus cells and mesenchymal stromal cells (MSCs) with the right growth factors. Combining these growth factors with the Collagen Meniscus Implant (CMI®) could accelerate cell ingrowth and tissue formation in the implant and thereby improve clinical outcomes. Using a transwell migration assay and a micro‐wound assay, the effect of insulin‐like growth factor‐1, platelet‐derived growth factor (PDGF), vascular endothelial growth factor (VEGF), transforming growth factor beta 1 (TGF‐*β*1), fibroblast growth factor, and platelet lysate (PL) on migration and proliferation of meniscus cells and MSCs was assessed. The formation of extracellular matrix under influence of the above‐mentioned growth factors was assessed after 28 days of culture of both MSCs and meniscus cells. As a proof of concept, the CMI® was functionalized with a VEGF binding peptide and coated with platelet‐rich plasma (PRP) for clinical application. Our results demonstrate that PDGF, TGF‐*β*1, and PL stimulate migration, proliferation, and/or extracellular matrix production of meniscus cells and MSCs. Additionally, the CMI® was successfully functionalized with a VEGF binding peptide and PRP which increased migration of meniscus cell and MSC into the implant. This study demonstrates proof of concept of functionalizing the CMI® with growth factor binding peptides. A CMI® functionalized with the right growth factors holds great potential for meniscus replacement after partial meniscectomy.

## 1 INTRODUCTION

The meniscus is a c‐shaped structure in the tibiofemoral joint composed of fibrocartilage. It is essential for load transmission, stability, and articular surface protection in the knee joint (Fox et al., 2015; Masouros et al., 2008). Meniscus injury is common and strongly correlates with the development of early osteoarthritis (OA) (Englund et al., 2009; Mitchell et al., 2016; Verdonk et al., 2016). Repair of meniscus injury is only successful in the vascularized region of the meniscus of young patients, where some regenerative capacity is present. Regeneration does not occur in the inner zone and in older patients (Mauck & Burdick, 2015; Osawa et al., 2013; Starke et al., 2009). Therefore, treatment often consists of (partial) meniscectomy, which increases the contact pressure in the articular cartilage, eventually leading to degeneration (I. D. McDermott & Amis, 2006; Roemer et al., 2017). Meniscus replacement or stimulation of meniscus regeneration could potentially prevent or delay the onset of OA (I. McDermott, 2011). A clinically available implant for partial meniscus replacement is the Collagen Meniscus Implant (CMI®; Stryker). The CMI® improves short‐term outcomes, but tissue deposition is limited and (partial) resorption occurs in several years (Schenk et al., 2020; Zaffagnini et al., 2007). In order to improve quality of the tissue and durability, a scaffold or implant could be seeded with cells (Korpershoek et al., 2017). Implantation of a scaffold seeded with autologous multipotent mesenchymal stromal cells (MSCs) has shown promising results in vivo*,* but has a high patient burden due to the necessity of two procedures (Desando et al., 2016; Hatsushika et al., 2014; Kondo et al., 2016). Moreover, harvesting and culturing of autologous cells are costly and time‐consuming; therefore, single‐stage procedure is highly preferable. In order to obtain a sufficient amount of cells in a single arthroscopic procedure without cell expansion, autologous meniscus cells from the meniscectomized tissue could be complemented with allogeneic MSCs (de Windt et al., 2017; Hagmeijer et al., 2019). Alternatively, incorporating growth factors within the scaffold could attract the patient's resident meniscus cells and MSCs present in the synovium and the meniscus (Segawa et al., 2009; Shariatzadeh et al., 2019; Sivasubramaniyan et al., 2019). The combination of growth factors present in platelet‐rich plasma (PRP) and platelet lysate (PL) were shown to have a positive effect on migration and proliferation of meniscus cells and MSCs (Freymann et al., 2016). To date, the effect of the different growth factors on migration of meniscus cells and MSCs remains to be elucidated. Furthermore, the in vivo lifespan of growth factors is too short to sustain biological activity (Ishida et al., 2007). Thus, a method to secure an ongoing effect is necessary. Such method has recently been described by Crispim et al., who immobilized growth factors on polycaprolactone (PCL) using a functionalization process for a growth factor binding peptide (J. Crispim et al., 2017; J. F. Crispim et al., 2018). However, it is still unknown whether this functionalization can be used to attract meniscus cells and MSCs to the CMI®.

Therefore, this study aims to assess the effect of the anabolic growth factors (1) insulin‐like growth factor‐1 (IGF‐1), (2) platelet‐derived growth factor (PDGF), (3) vascular endothelial growth factor (VEGF), (4) transforming growth factor beta 1 (TGF‐*β*1), (5) fibroblast growth factor (FGF), and PL on migration, proliferation, and extracellular matrix (ECM) production of meniscus cells and MSCs. Moreover, it shows proof of concept of the functionalization of the CMI® with a growth factor binding peptide and assesses the effect of this functionalization. We hypothesize that these growth factors and PL accelerate meniscus regeneration by targeting the mechanisms mentioned above. Additionally, we hypothesize that by functionalization of the CMI® with a growth factor binding peptide, a continued effect of the targeted growth factor could be achieved.

## 2 MATERIALS AND METHODS

### 2.1 Cell isolation

Human meniscus cells were isolated from menisci from patients who had undergone total knee arthroplasty. Collection of meniscus tissue was performed according to the Medical Ethics regulations of the University Medical Center Utrecht and the guideline “Human Tissue and Medical Research: Code of Conduct for responsible use” of the Dutch Federation of Medical Research Societies (Federa, 2011; van Diest, 2002). The menisci were washed in phosphate‐buffered saline (PBS) twice and manually cut into pieces of 2 mm. The tissue was digested in 0.15% collagenase type II (CLS‐2, Worthington) in DMEM (Gibco, Life Technologies) with penicillin (100 U/ml; Gibco, Life Technologies) and streptomycin (100 mg/ml; Gibco, Life Technologies) (1% pen/strep), at 37°C overnight. Meniscus cells were expanded in DMEM supplemented with 10% fetal bovine serum (FBS; HyClone) and 1% pen/strep and used at passage 2.

The use of human MSCs was approved by the institutional ethical review board (TCBio 08‐001 and 18/739). MSCs were isolated from bone marrow aspirates obtained from three donors who provided written informed consent. MSCs were isolated and characterized as described previously (Gawlitta et al., 2012), and expanded in αMEM supplemented with 10% FBS (HyClone), 0.2 mM l‐ascorbic acid‐2‐phosphate (2% ASAP, Sigma‐Aldrich), and 1% pen/strep for use at passage 3.

### 2.2 Growth factors and platelet lysate medium

Human recombinant IGF‐I (Sigma‐Aldrich), human PDGF (Sigma‐Aldrich), human recombinant FGF‐basic (R&D Systems), human recombinant TGF‐*β*1 (R&D systems), and human recombinant VEGF (Novus Biologicals) were diluted in concentrations of 10, 1, and 0.1 ng/ml in Dulbecco's modified Eagle's medium (DMEM; Gibco, Life Technologies) supplemented with 2% Albuman (human serum albumin 200 g/l; Sanquin), 2% ASAP, 2% Insulin‐Transferrin‐Selenium‐X (ITSX, Invitrogen), and 1% pen/strep.

For the preparation of PRP and PL, blood was obtained through the Mini Donor Service, a blood donation facility for research purposes approved by the medical ethics committee of the University Medical Center Utrecht, The Netherlands. All donors have provided written informed consent, in accordance with the declaration of Helsinki. Whole blood was collected in 3.2% sodium‐citrate‐containing tubes and centrifuged at 250 G for 10 min. The pelleted erythrocytes were discarded and the top layer containing the platelets was centrifuged at 750 G for 10 min. The supernatant (plasma) was collected, and the pellet containing the platelets was suspended in one‐third of the supernatant. PL was formed by freeze‐thawing the suspension for three cycles (−80°C to 37°C) in order to release the growth factors from the platelets, and centrifuged at 8000 G for 10 min. Upon use, the PL was diluted at 1% and 10% in DMEM supplemented with 2% Albuman, 2% ASAP, 2% ITSX, 1% pen/strep, and 3.3 U/ml heparin. Using the CELL‐DYN Emerald hematology analyzer (Abbott B.V., Abbott Park, Illinois), PRP of nine donors was characterized in terms of platelet, erythrocyte, and leucocyte concentration. Pooled PRP of at least three donors was used in the experiments.

### 2.3 Micro‐wound assay

Both meniscus cells and MSCs (*n* = 3) were seeded in monolayer and expanded up to 80% confluency in a 12‐well plate. Cells were washed with PBS, a micro‐wound was made by scratching over the cell monolayer with a 200 μl pipette tip, and cell debris was aspirated after an additional wash with PBS. Growth factors and PL were diluted as mentioned above and supplemented with 10 μM 5‐ethylnyl‐2′‐deoxyuridine (EdU; Click‐iT™ EdU Alexa Fluor® 488 Imaging Kit; Invitrogen) and added to the wells. At 0, 24, and 48 h after scratching, six pictures were taken along the micro‐wound using an inverted light microscope. Using Photoshop CS6 software (Adobe Systems), the pictures were merged, and an area of 17.708 by 48.697 pixels was cropped out at the same spot for every time point, and analyzed in ImageJ. The cells in the scratch were identified using color thresholding and were calculated as percentage of the image at 0 h after scratching. After 48 h, cells were washed with PBS, fixated in formaldehyde 4% (Klinipath), and permeabilized with PBS‐Tween (PBST) 0.1%. Proliferated (EdU) and total cells (Hoechst) were visualized using the manufacturer's protocol using excitation and emission of 495/519 and 392/440 nm, respectively (Thermo Fisher Scientific). Three pictures were taken at different locations along length of the micro‐wound using an EVOS FLoid™ Cell Imaging microscope, and analyzed via color thresholding and “analyze particles” in ImageJ.

### 2.4 Transwell migration assay

Meniscus cells and MSCs were trypsinized and suspended in DMEM supplemented with 2% Albuman, 2% ASAP, 2% ITSX, and 1% pen/strep in a concentration of 5 × 10^5^ cells/ml. 450 μl of this cell suspension was added to the cell culture inserts (12 mm, polycarbonate, 8.0 μm; Merck Millipore) which were placed in a 24‐well plate. 450 μl of growth factor, PL, or control medium was added to the wells of the 24‐well plate. The plates were incubated for 4 h at 37°C, before washing with PBS, and cleaning the upside of the polycarbonate membrane with a Q‐tip to remove the remaining cells. Cells that were migrated through the membrane were fixated in formaldehyde 4%, and stained using Mayer's Hematoxylin. The polycarbonate membrane was cut out of the insert, mounted on a microscope slide, and migrated cells were counted using a light microscope.

### 2.5 Extracellular matrix formation

Meniscus cells were resuspended in a 1:15 diluted fibrinogen component of Tisseel fibrin glue (Baxter international Inc.) and combined with 1:50 diluted thrombin (Tisseel, Baxter international Inc.). The fibrin constructs consisting of 2.5 × 10^5^ cells in 100 μl were allowed to gelate for 15 min at 37°C. Afterwards, the constructs were put in a 48‐well plate with 250 μl growth factor, PL, or control medium. The fibrin constructs were cultured for 28 days at 37°C with 5% CO_2_, medium was changed three times per week, and conditioned medium was stored at −20°C for analysis.

### 2.6 Functionalization of the Collagen Meniscus Implant for different growth factors

For functionalization of the CMI®, peptides with the sequence KGSWWAPFH (VEGF binding peptide) and KGSWWSSSH (scrambled peptide) were synthesized following Fmoc solid peptide synthesis procedures as described previously (J. Crispim et al., 2017), purified and characterized with high‐performance liquid chromatography (HPLC) and mass spectrometry. The CMIs® were incubated in 1 ml 50 mM 2‐(N‐morpholino)ethanesulfonic acid (MES) buffer (pH = 5.2) containing 1 mM of peptide during 1 h at room temperature. After 1 h, 1 ml of MES containing 50 mM of N‐hydroxysuccinimide/1‐ethyl‐3‐(3‐dimethylaminopropyl)‐carbodiimide (NHS/EDC) was added to the CMIs®. The reaction was carried out for 24 h at room temperature. The functionalized CMIs® were washed three times with PBST (0.5%), and afterwards rinsed three times with PBS (Ma et al., 2004).

Using this method, five different conditions of functionalization were created to determine the quality of the functionalization: (1) CMI® + MES buffer, without VEGF, (2) CMI® + MES buffer, with VEGF, (3) CMI® + EDC/NHS and MES buffer and VEGF, and (4) CMI® + EDC/NHS + Scrambled VEGF Peptide and VEGF, and (5) CMI® + EDC/NHS + VEGF binding peptide and VEGF.

After functionalizing the CMIs® for the VEGF binding peptide, they were incubated with 1 μg/ml of VEGF (PeproTech) in PBST 0.5% for 1 h with gentle agitation. Afterwards, the CMIs® were washed three times for 10 min with PBST 0.5% and PBS, and blocked for 1 h with PBS containing 1% (w/v) bovine serum albumin (BSA) followed by the same washing steps. For imaging, CMIs® were incubated with a primary antibody (2 μg/ml; rabbit polyclonal anti‐human VEGF, PeproTech) in the blocking solution for 1 h with agitation. The CMIs® were washed as mentioned above and incubated with a secondary antibody (8 μg/ml; goat anti‐rabbit Alexa Fluor 594, Invitrogen) in PBS containing 1% w/v BSA for 1 h with gentle agitation. Before image acquisition with a fluorescence microscope, the CMIs were washed three times for 10 min with PBST and rinsed three times with PBS. Fluorescence intensity was quantified using ImageJ. PRP was used to coat the CMI®. First, 40 μl of PRP was added to the CMI® slices, followed by 20 μl of CaCl_2_ and 20 μl of thrombin (Tisseel, Baxter). The constructs were incubated for 15 min at 37°C to allow gelation of the PRP gels in the CMI®.

### 2.7 Cell migration into the functionalized Collagen Meniscus Implant

Cell migration into CMI® assays were conducted in four groups: (1) VEGF‐functionalized, (2) scrambled peptide, (3) PRP‐functionalized, and (4) control (non‐functionalized). Meniscus cells and MSCs were trypsinized incubated for 1 h at 37°C in lipophilic live cell membrane stain Vibrant CM‐DiI. Fibrin constructs with 2.5 × 10^5^ cells (meniscus or MSCs) were formed as described above, which were seeded onto the outer rim of the cross section of the four groups of CMIs®. The constructs were cultured for 7 days in DMEM supplemented with 2% Albuman, 2% ASAP, 2% ITSX, Invitrogen, and 1% pen/strep, and medium was changed every other day.

### 2.8 Confocal microscopy imaging

After 7 days of culture, the CMIs® were washed three times for 10 min with PBST and rinsed three times with PBS to remove the unattached cells. The CMI® was counterstained with DAPI for 4 min the CMIs® were imaged using confocal microscopy (Leica SP8X) using excitation at 358 and 549 nm. Images of the two channels were merged using ImageJ, and color threshold was applied to select cell area and collagen area. Selected cell area and CMI® collagen fibers area were used to calculate the total area with cells per collagen fibers.

### 2.9 VEGF retention

For analyzation of VEGF retention by the CMI® and release into the medium, CMIs® were incubated with 100 ng VEGF (Novus Biologicals), washed six times in PBST and PBS and subsequently cultured for 7 days in DMEM supplemented with 2% Albuman, 2% ASAP, 2% ITSX, Invitrogen, and 1% pen/strep. Culture medium was changed every other day. VEGF concentrations in the media and washing fluid were determined using an enzyme‐linked immunosorbent assay (ELISA) according to the manufacturer's instructions (Duoset ELISA kit, R&D Systems).

### 2.10 Biochemical analyses

Biochemical analyses were performed on fibrin constructs cultured for assessment of ECM formation and the functionalized CMIs®. After culturing, the fibrin constructs and functionalized CMIs® were digested at 60°C overnight in papain buffer (250 µg/ml papain (Sigma‐Aldrich), 0.2 M Na2EDTA, 0.1 M NaAc, and 0.01 M cysteine). The PicoGreen® dsDNA quantitation assay was used according to the manufacturer's instructions to determine the DNA content of the constructs. Excitation was set at 480 nm, emission 520 nm, and λDNA was used as a standard reference. Glycosaminoglycan (GAG) content was determined using dimethylmethylene blue (DMMB) assay. Chondroitin sulfate (Sigma‐Aldrich) was used as standard and absorbance measured at 525 and 595 nm.

Papain samples were both freeze‐dried and hydrolyzed overnight at 108°C for determining collagen content using a hydroxyproline assay. Chloramine‐T (Merck) and dimethylaminobenzoaldehyde (Merck 3058) were added, and hydroxyproline (Merck 104506.0010) was used as standard to measure the hydroxyproline content at 570 nm. Collagen content was calculated from the hydroxyproline content, since 13.5% of collagen is composed of hydroxyproline (Neuman & Logan, 1950).

### 2.11 Statistical analyses

Statistical analyses were performed using GraphPad Prism 7 (GraphPad Software, Inc.). Data are presented as mean ± SD. A two‐way analysis of variance (ANOVA) and the Dunnett post‐hoc test were performed to determine significant differences between all growth factor or PL groups and the control, and the interactive effect of donor variability was taken into account.

Confocal images for cell ingrowth into the CMI® were analyzed using ImageJ. Student *t*‐test was used to assess the significance level of difference between VEGF‐functionalized groups and scrambled‐peptide groups; and PRP‐functionalized group with non‐functionalized group. ANOVA was used to assess the difference between time points. *p*‐Values <0.05 were considered statistically significant.

## 3 RESULTS

### 3.1 PRP characterization

Compared to whole blood, PRP contained 1.5 times concentrated platelets (298.5 ± 166.3 × 10^9^/L). PRP contained 1.2 ± 0.9 × 10^9^/L leucocytes and 23.3 ± 12.2 × 10^9^/L erythrocytes.

### 3.2 PDGF and platelet lysate increase migration of meniscus cells and MSCs

#### 3.2.1 Micro‐wound

**FIGURE 1:**
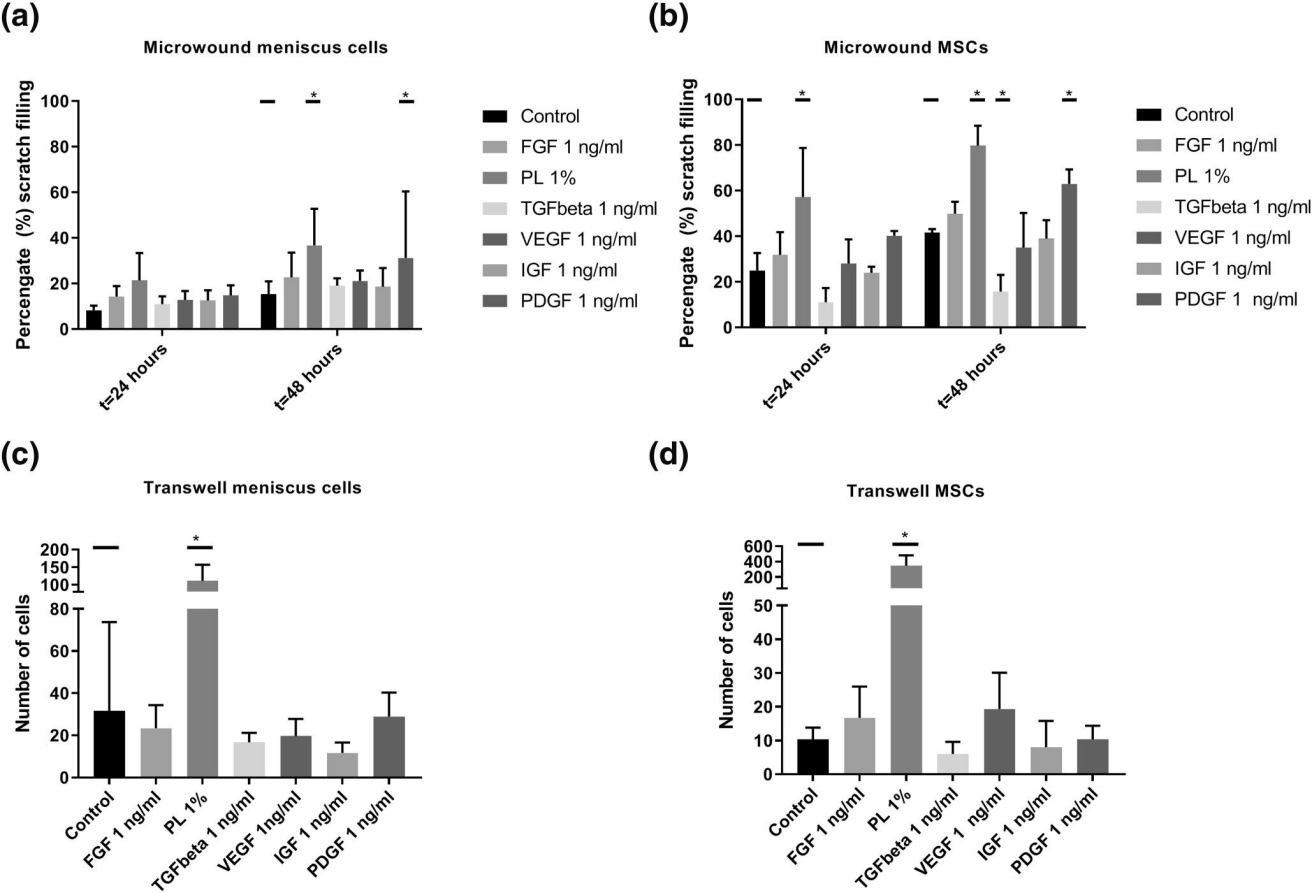
Migration of meniscus cells and mesenchymal stroma cells (MSCs) in the micro‐wound assay (A and B) and the transwell assay (C and D) using fibroblast growth factor (FGF), platelet lysate (PL), transforming growth factor beta 1 (TGF‐*β*1), vascular endothelial growth factor (VEGF), insulin‐like growth factor‐1 (IGF‐1), and platelet‐derived growth factor (PDGF). *p < 0.05

Filling of the micro‐wound by meniscus cells in medium supplemented with 10.0, 1.0, and 0.1 ng/ml of growth factors or 1 or 10% of PL was evaluated at 24 and 48 h. At 24 h, the wound filling in the 10% PL conditions was significantly higher (*p* < 0.0001) compared to the control group (Figure S1). At 48 h, 1% PL (36.7 ± 16.1% filling) and 1 ng/ml PDGF (31.1 ± 29.2% filling) significantly increased wound filling compared to the control (15.3 ± 5.6%). Wound filling of VEGF and FGF did not reach statistical significance (Figure 1a). In general, at 48 h, the presence of growth factors at a concentration of 1 ng/ml led to more wound filling compared to the other concentrations (Figure S1). Therefore, all other experiments were continued with a growth factor concentration of 1 ng/ml and 1% PL.

Compared to the meniscus cells, wound filling was more extensive in the MSCs. 1% PL significantly increased wound filling of MSCs compared to the control (57.2 ± 21.6% compared to 24.0 ± 2.7%) at 24 h. At 48 h, both 1% PL and PDGF showed an increased filling of the scratch with 79.7 ± 8.8%, and 62.9 ± 6.4%, compared to 41.5 ± 1.6% for the control. TGF‐*β*1 decreased wound filling compared to the control at 48 h, with only 15.7 ± 7.3% coverage of the scratch. Other GFs did not increase or decrease wound filling (Figure 1b).

### 3.3 Transwell migration

PL significantly increased migration of both meniscus cells and MSCs in the transwell migration assay. For meniscus cells 1% PL significantly increased the number of migrated cells from 12 ± 9 to 111 ± 46 (Figure 1c). For MSCs, the number of migrated cells in the control group was 10 ± 4 compared to 346 ± 137 in the 1% PL group (*p*‐value <0.01) (Figure 1d). PDGF and FGF did not significantly increase meniscus cell migration, and VEGF and FGF did not increase migration in MSCs.

### 3.4 TGF‐*β*1 and platelet lysate increased proliferation of meniscus cells

**FIGURE 2:**
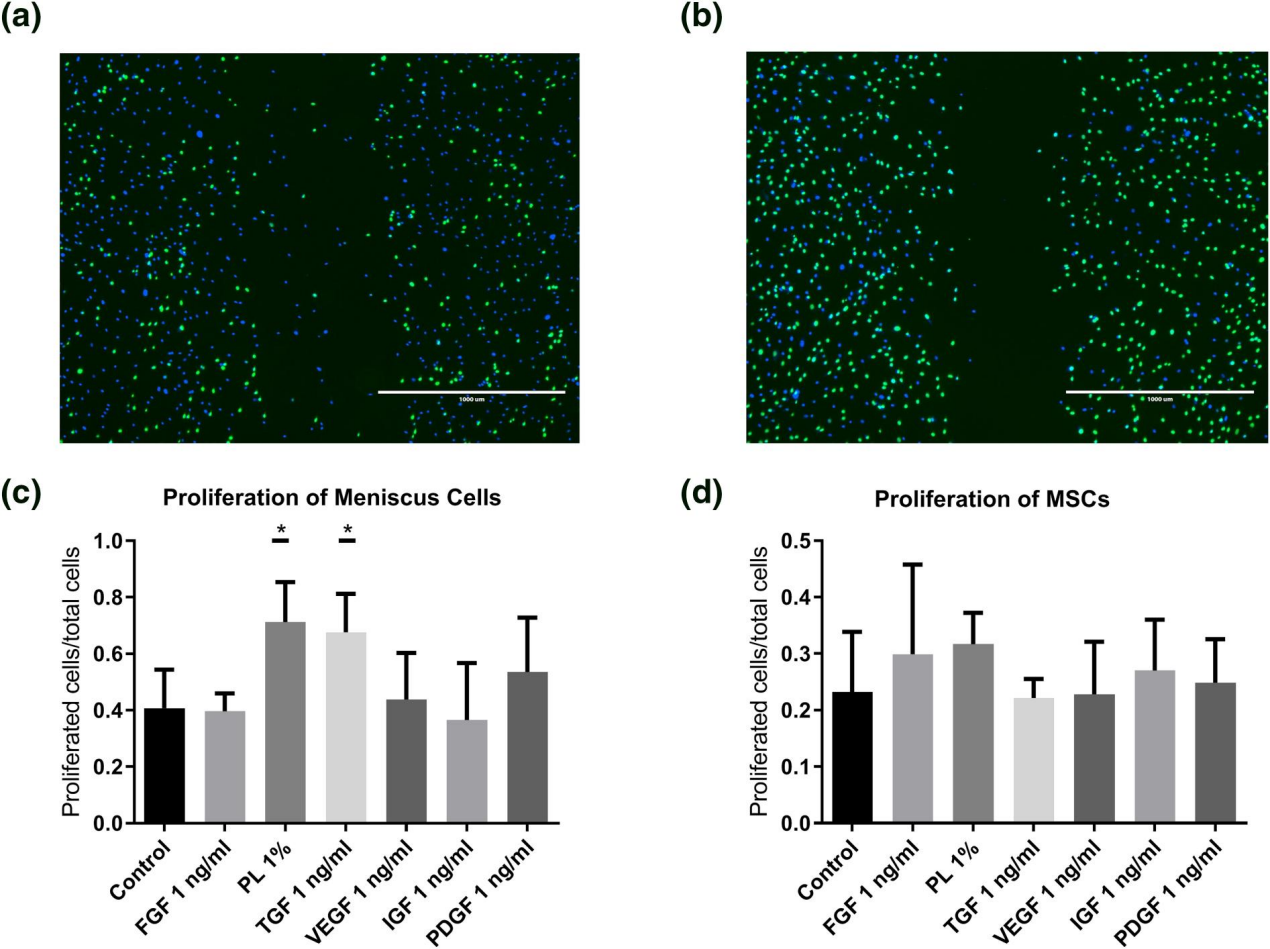
Proliferation of meniscus cells and mesenchymal stromal cells (MSCs) using fibroblast growth factor (FGF), platelet lysate (PL), transforming growth factor beta 1 (TGF‐*β*1), vascular endothelial growth factor (VEGF), insulin‐like growth factor‐1 (IGF‐1), and platelet‐derived growth factor (PDGF) in the micro‐wound assay, demonstrated by 5‐ethynyl‐2′‐deoxyuridine (EdU) assay. Proliferated cells shown in green by the EdU staining and the non‐proliferated cells are stained blue using Hoechst. An example from the control (A) compared to TGF‐*β*1 group after 48 h (B). Proliferated cells/total cells for meniscus cells (C) and MSCs (D). *p < 0.5

By labeling the proliferated cells with EdU in the micro‐wound assay, the ratio of proliferated cells/total amount of cells (green/blue ratio) at 48 h was calculated (Figure 2a,b). For meniscus cells, the control group showed a ratio of 0.41 ± 0.14, which was significantly lower than the 0.71 ± 0.14 ratio of PL and 0.68 ± 14 in TGF‐*β*1. The proliferation was not significantly higher in the PDGF group (0.54 ± 0.19) compared to the control group (Figure 2c). Overall, MSCs showed a lower proliferation ratio compared to the meniscus cells. Besides, none of the growth factors or PL significantly increased the proliferation of MSCs after 48 h (Figure 2d).

### 3.5 TGF‐*β*1 stimulates production of extracellular matrix of meniscus cells

**FIGURE 3:**
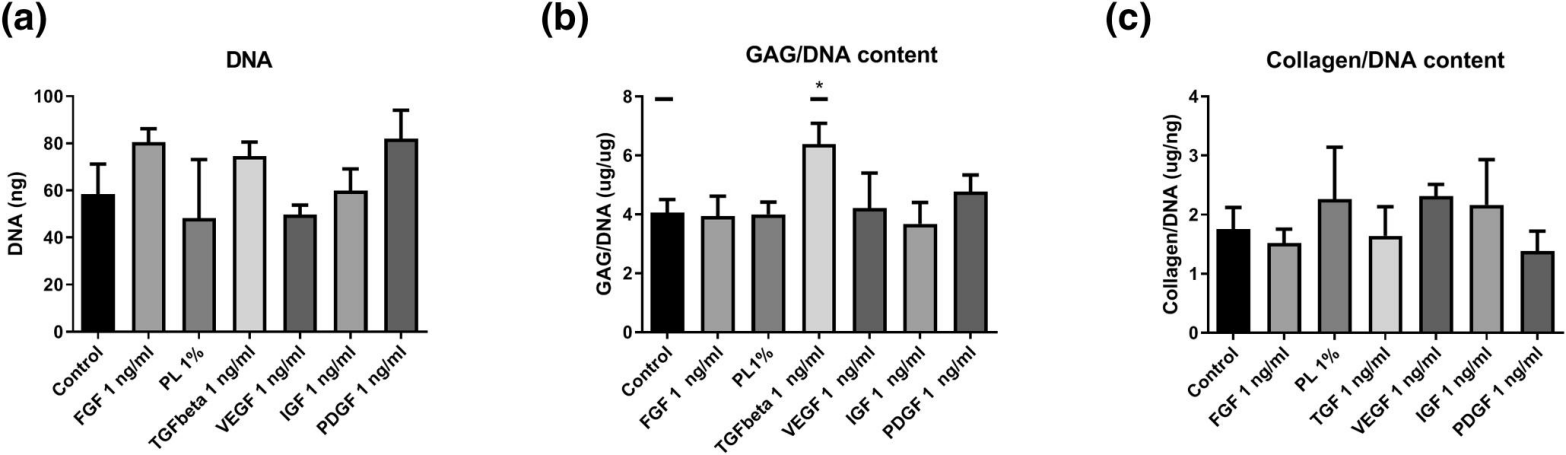
Biochemical analysis after 28 days of culturing meniscus cells in fibrin glue constructs with the addition of fibroblast growth factor (FGF), platelet lysate (PL), transforming growth factor beta 1 (TGF‐*β*1), vascular endothelial growth factor (VEGF), insulin‐like growth factor‐1 (IGF‐1), and platelet‐derived growth factor (PDGF). Figure shows DNA content (A), glycosaminoglycan content normalized for DNA (GAG/DNA) (B), and collagen content normalized for DNA (C). *p < 0.05

The DNA content of the different constructs did not differ significantly after 28 days of culture; however, there was a trend toward a higher DNA content in FGF, TGF‐*β*1, and PDGF compared to the control group (Figure 3a). TGF‐*β*1 significantly increased formation of GAGs in the fibrin glue constructs (Figure 3b). There was no significant effect of any of the growth factors or PL on the production of collagen (Figure 3c)

### 3.6 Functionalization of the Collagen Meniscus Implant increases the cell ingrowth

**FIGURE 4:**
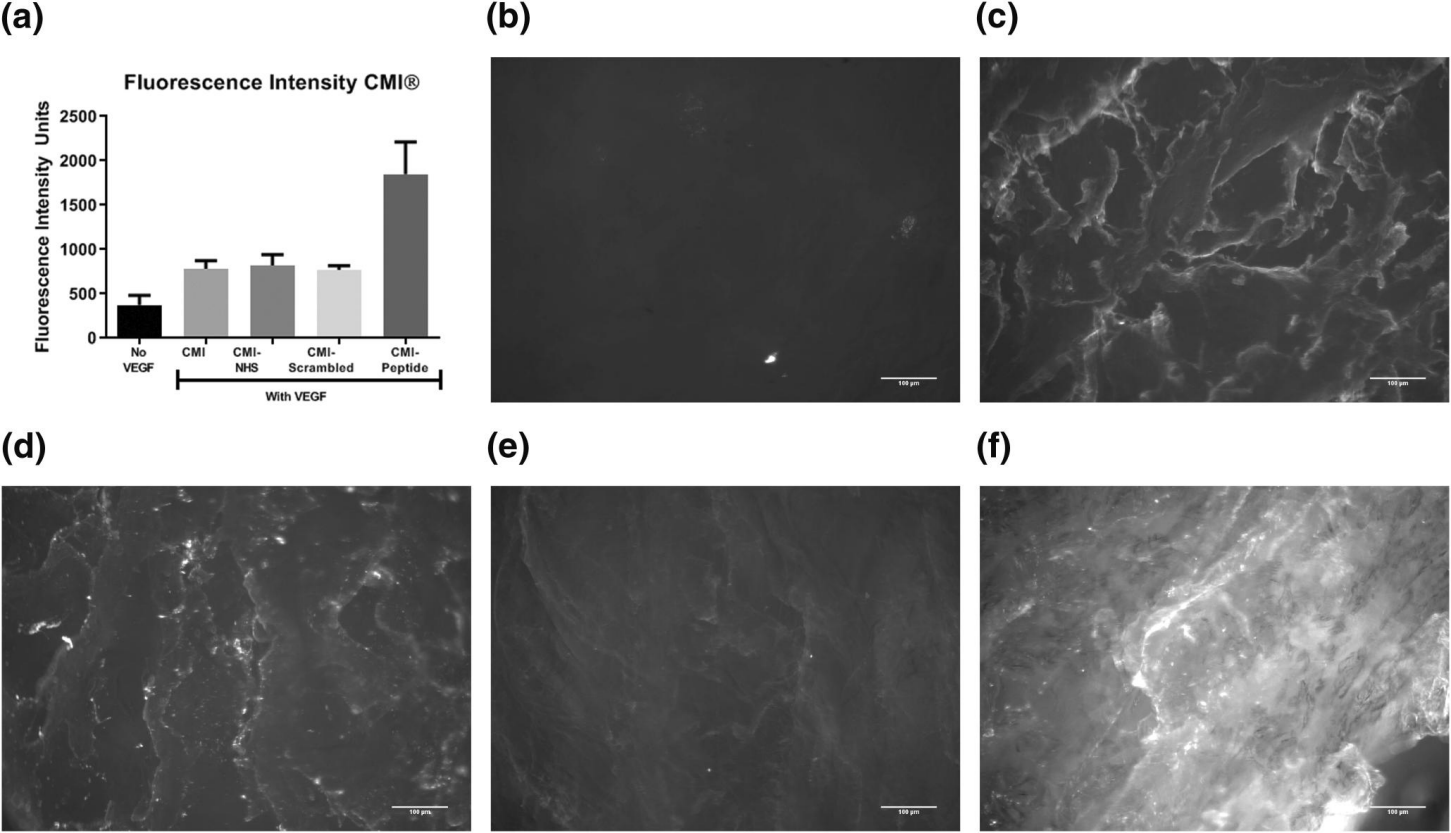
Immobilization of vascular endothelial growth factor (VEGF) on functionalized Collagen Meniscus Implants (CMI®) using a VEGF binding peptide (*n* = 8). Quantified fluorescence intensity (A). Example images of CMI® with MES buffer without VEGF (B), CMI® with MES buffer and VEGF (C), CMI® with EDC/NHS, MES buffer and VEGF (D), CMI® with EDC/NHS, a scrambled VEGF peptide and VEGF (E), and CMI® with EDC/NHS, VEGF binding peptide and VEGF (F)

The CMI® was successfully functionalized with VEGF binding peptide. Figure 4 shows fluorescence microscopy images of the five different groups of functionalized CMI, with fluorescently labeled VEGF bound to the CMI®. Compared to the four other groups (Figure 4b–e), the CMI® functionalized with the VEGF binding peptide (Figures 4f and S2) showed significantly higher fluorescence intensity units. Moreover, VEGF binding was 94.8 ± 1.4% in the CMI® functionalized for VEGF, compared to almost no binding in the CMI® functionalized for scrambled peptide (Figure S3a). VEGF release in medium was lower in the CMI® functionalized for VEGF, than the CMI® functionalized for scrambled peptide and plain CMI® (Figure S3b).

**FIGURE 5:**
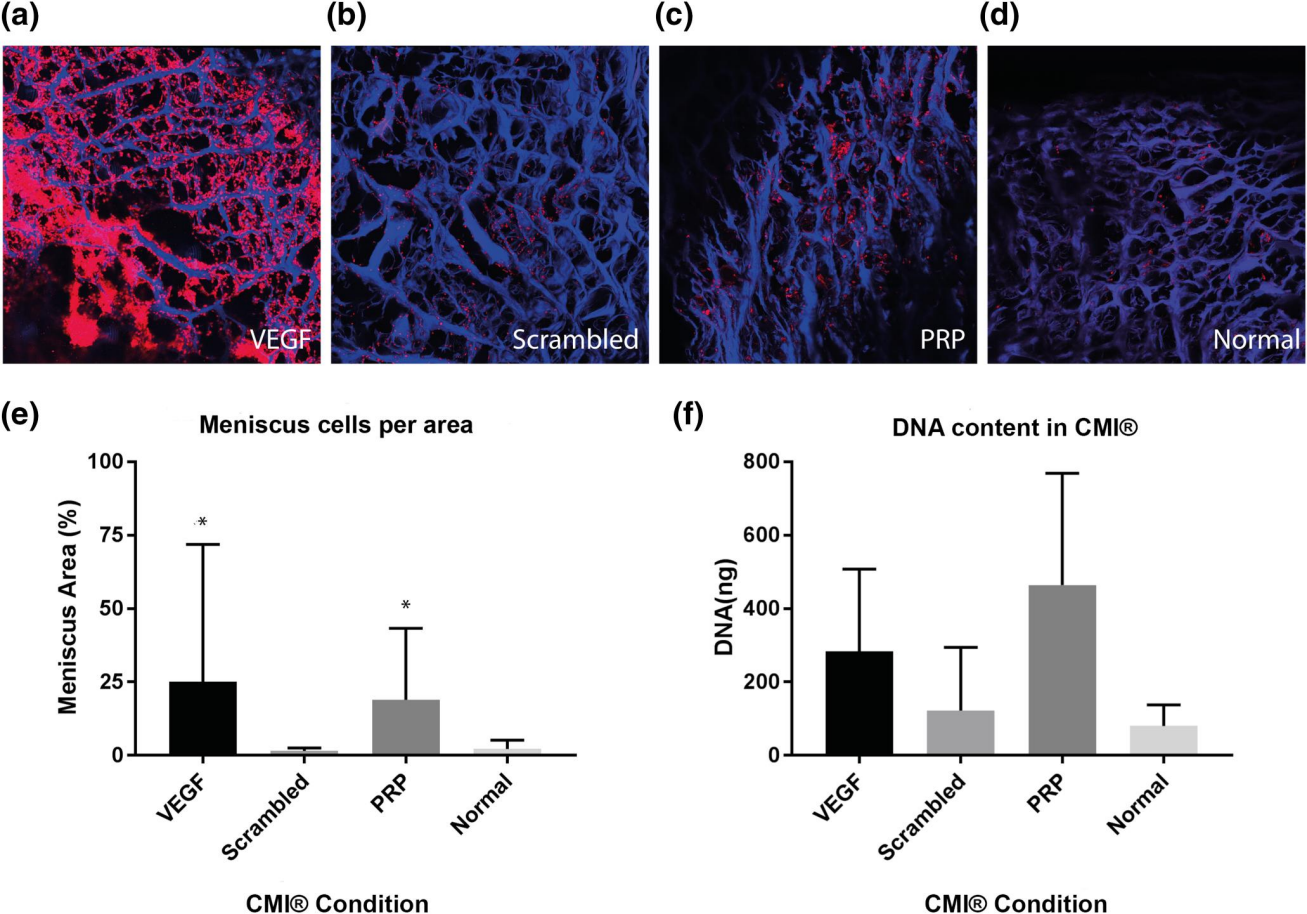
Cell migration of meniscus cells into the Collagen Meniscus Implant (CMI®) (*n* = 6). The CMI® is stained with DAPI (blue) and meniscus cells with DiI (red). The figure shows CMI® with EDC/NHS, VEGF peptide and VEGF (A), and CMI® with EDC/NHS, a scrambled VEGF binding peptide and VEGF (B), CMI® coated with PRP (C) and CMI® (D). E corresponds with A–D, and shows percentage of meniscus cells per CMI® area. F shows DNA quantification in the whole constructs. *p < 0.05

**FIGURE 6:**
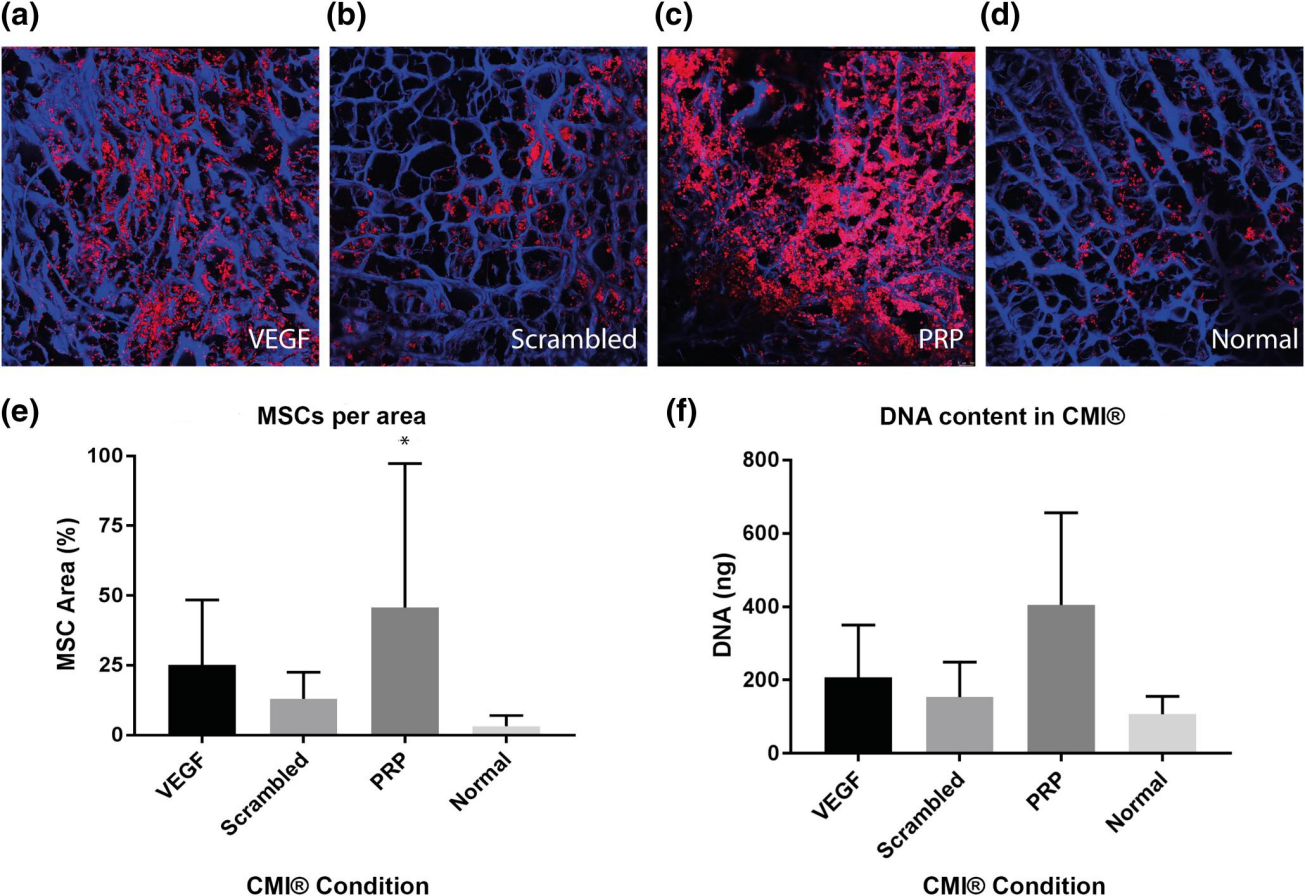
Cell migration of mesenchymal stromal cells (MSCs) into the Collagen Meniscus Implant (CMI®) (*n* = 6). The CMI® is stained with DAPI (blue) and MSCs with DiI (red) (A–D). The figure shows CMI® with EDC/NHS, VEGF peptide and VEGF (A), and CMI® with EDC/NHS, a scrambled VEGF binding peptide and VEGF (B), CMI® coated with PRP (C) and CMI® (D). E corresponds with A–D, and shows percentage of meniscus cells per CMI® area. F shows DNA quantification in the whole constructs. *p < 0.05

In the migration assay with constructs of meniscus cells in fibrin glue, significantly more meniscus cells were present in the CMIs® functionalized for VEGF and coated with PRP after 7 days. In VEGF‐functionalized groups, the meniscus cells were aligned well along the CMI® fibers, and showed cell aggregates in the higher cell density areas (Figure 5a). In PRP groups, cells were situated along fibers and the space between fibers filled with PRP gels, compared to round cells not aligned along the fibers in the scrambled and negative control group (Figure 5b–d). Similar effects were seen for MSCs (Figure 6a–d). Comparison of the area of meniscus cells and MSCs standardized for area of CMI® collagen fibers between conditions are shown in Figures 5e and 6e. A significant difference can be observed between VEGF‐functionalized group and scrambled‐peptide functionalized group for meniscus cells migration. A significant difference between PRP‐coated group and negative control was also observed in both meniscus cell and MSC migration. The density of MSCs in the PRP‐functionalized group did not differ significantly compared to VEGF‐functionalized group.

DNA quantification after papain digestion showed results in accordance with the confocal pictures and analysis of the images. The highest cell amounts were seen in the PRP and VEGF group, followed by the scrambled peptide and the negative control (Figures 5f and 6f), although the differences did not reach statistical significance.

## 4 DISCUSSION

Regeneration or replacement of the meniscus can potentially prevent or delay onset of osteoarthritis after meniscectomy. In the current study, we explored the potential of several growth factors and PRP/PL for stimulating the migration, proliferation, and ECM formation of meniscus cells and MSCs. Moreover, we demonstrated proof of concept of a technique to capture and immobilize growth factors to a clinically available meniscus implant for the purpose of increased regeneration after meniscus replacement.

We based the choice of growth factors and concentrations on previous literature on meniscus cells, cartilage, and chondrocytes (Bhargava et al., 1999; Forriol, 2009; Petersen et al., 2007). As a wide range of dose‐dependent concentrations of growth factors is given in literature (Bhargava et al., 1999; Imler et al., 2004), the concentration we used was based on results of a pilot study (Figure S1) with concentrations of 0.1, 1, and 10 ng/ml for the growth factors, and concentrations 0.1%, 1%, and 10% for PL. We were reluctant to use high growth factor concentrations, as overdosing is a general concern for regenerative therapies (James et al., 2016).

In the transwell and micro‐wound assay, migration of both meniscus cells and MSCs was increased by PL and PDGF. The effect of PL on migration of meniscus cell was similar to the effect described by Ishida et al. (2007), who demonstrated that migration of meniscus cells and synovial MSCs into a biodegradable gelatin hydrogel increased by addition of PRP. Similarly, migration into a decellularized meniscus increased by PDGF‐BB bound to the scaffolds (Lee et al., 2018) and transwell migration of murine MSCs increased by PDGF‐AA (Li et al., 2014). In the current study, migration of MSCs in the transwell assay decreased by TGF‐*β*1. This decreased migration could be attributed to the stimulatory effect of TGF‐*β*1 for chondrogenic differentiation of MSCs, which might in turn inhibit migration and proliferation. However, migration in a transwell assay in murine MSCs did increase by TGF‐*β*1 (Dubon et al., 2018) at a concentration of 100 ng/ml, which might indicate a dose‐dependent effect. Additionally, in the micro‐wound assay, proliferation of meniscus cells increased by PL and TGF‐*β*1. However, TGF‐*β*1 had no significant effect on proliferation in the study by Riera et al. (2011), which could be explained by the fact that they used pig cells, which might respond different to human recombinant TGF‐*β*1 than the human cells that were used here. The effect of TGF‐*β*1 should be further elucidated in order to clear up these controversies. Interestingly, proliferation of MSCs was not stimulated by any of the growth factors, although VEGF, PDGF, TGF‐*β*1, and FGF were previously reported to increase proliferation (Rodrigues et al., 2010). These differences could indicate a dose‐dependent effect of the growth factors. In the current study, the proliferation rate of MSCs was lower compared to meniscus cells, as assessed by the proportion of cells that have proliferated at least once. It has been described that in MSCs, a small proportion of the cells divides rapidly and is responsible for the high proliferation rate in the total population. ECM formation significantly increased by the addition of TGF‐*β*1 in the medium of meniscus cells. Indeed, increased proteoglycan synthesis of meniscus cells on scaffolds in presence of TGF‐*β*1 and FBS have been described earlier (Gunja et al., 2009). Matrix formation was not stimulated by PL in our cultures, similar to the deleterious effect of PL addition during the redifferentiation phase in cartilage (Rikkers et al., 2020). The stimulatory effects of PL, PDGF, and TGF‐β1 on migration, proliferation, and/or ECM formation identify these growth factors (substrates) as potential target for meniscus regeneration. Targeting migration, proliferation, and ECM production at the same time by different growth factor hold great potential, as this could work as a catalyst. Indeed, the addition of PDGF to TGF‐*β*1 led to a threefold increase in collagen production compared to the use of TGF‐*β*1 alone (Hoben et al., 2009). The combined effect of these growth factors should be further investigated.

In the current research, proof of concept of functionalization of the CMI® with growth factor binding peptides was demonstrated. Functionalization with a PDGF binding peptide holds promise as shown in the migration and proliferation assays. However, PDGF has three different isoforms (AA, AB, and BB) affecting different PDGF receptors (Lepisto et al., 1995), which makes it unfavorable to use PDGF in this proof‐of‐concept study. PDGF and VEGF family members are closely related (Ball et al., 2007); therefore, functionalization with a VEGF capturing peptide was chosen for this proof‐of‐principle study. Peptides for VEGF binding demonstrate that a variety of growth factors can be used for functionalization. Due to the stimulatory effect of PL on migration and proliferation, a PRP‐coated CMI® was also as a proof of concept. Both the CMI® functionalized for VEGF and the CMI® coated with PRP resulted in a higher cell density inside the CMI® after 7 days of culture compared to the negative control. The role of PRP for regenerative strategies in cartilaginous tissues remains controversial, as PRP seems to primarily increase proliferation, while at the same time decreasing differentiation (Rikkers et al., 2020). In vivo, PRP could provide an initial boost for proliferation, and after the rapid decline in growth factors concentration due to the short half‐life, redifferentiation and ECM formation could occur. The CMI® functionalized for VEGF and the CMI® coated with PRP show high potential for clinical translation by the attraction of endogenous growth factors present in the knee joint without injecting additional growth factors.

There are limitations of the current study design. First, the effect of single growth factors was examined in this study, whereas functionalization of the CMI® with multiple growth factor binding peptides is possible, and therefore, combinations of two or more growth factors should be further investigated. Based on the results in this study, the potential effect of PDGF in combination with TGF‐*β*1 should be further explored. Secondly, PL and PRP contain a mix of growth factors, and the effect of PRP and PL can therefore be attributed to an additional effect of the growth factors present. More insight into the effects of PRP and PL could in the future be obtained by testing a “synthetic PRP” using a combination of growth factors in their concentrations as present in the PRP. Thirdly, the effect of the functionalization on mechanical properties of the CMI® was not investigated. However, we do not expect functionalization to have a major impact on the mechanical properties in the short term, as the collagen network in the CMI® is not disrupted by the functionalization process. In the long term, we expect of positive effect of the functionalization due to improved tissue formation, which should be confirmed in vivo. Moreover, we expect no decrease in quality of functionalization upon implantation as there was no negative effect of mechanical stress on the functionalization of bioactive tape or polycaprolactone for other growth factors in vivo (J. Crispim et al., 2017; J. F. Crispimet al., 2018). Additionally, the stability of the functionalization is based on highly stable amide bond (Ward et al., 2010) and amidation and acetylation of the peptides which makes them resistant to proteolytic and enzymatic degradation (Nguyen et al., 2010). Lastly, a disadvantage of the CMI® is that does not restore the exact composition, morphology and mechanical characteristics of the native meniscus, but it is one of the few implants that is currently available in the clinic. Functionalization of the CMI® might lead to formation of native meniscus tissue and overcome these disadvantages.

In conclusion, this study demonstrated stimulation of migration, proliferation, and/or ECM production for meniscus cells and MSCs using PDGF, TGF‐*β*1, and PL. Additionally, the CMI® was successfully functionalized with a VEGF binding peptide and PRP which led to increased meniscus cell and MSC migration into the meniscus implant. Therefore, the results of this study demonstrate feasibility of functionalization of the CMI® with growth factor binding peptides or PRP for enhancement meniscus regeneration after partial meniscectomy.

## CONFLICT OF INTEREST

None of the authors have declared a conflict of interest regarding this manuscript.

## AUTHOR CONTRIBUTIONS

Michella H. Hagmeijer, Jasmijn V. Korpershoek, Aaron J. Krych, Daniel B.F. Saris, and Lucienne A. Vonk designed the study protocol. Michella H. Hagmeijer, Jasmijn V. Korpershoek, Lucienne A. Vonk, and Lucienne A. Vonk performed the experiments. Michella H. Hagmeijer and Jasmijn V. Korpershoek analyzed data and wrote the manuscript; João F. Crispim and Pascal Jonkheijm planned and performed CMI® functionalization. All authors interpreted data, reviewed previous versions of the manuscript and read and approved the final manuscript.

## Supporting information

Supplementary Material 1

## Data Availability

The data that support the findings of this study are available from the corresponding author upon reasonable request.
