# Supplementary material for: The regenerative effect of different growth factors and platelet lysate on meniscus cells and mesenchymal stromal cells and proof of concept with a functionalized meniscus implant
Source: J Tissue Eng Regen Med. 2021 May 21;15(7):648–59. doi: 10.1002/term.3218 (PMC8362003; doi:10.1002/term.3218)
Supplement: Supplementary file 1 — Supplementary Material 1 [file TERM-15-648-s001.docx]

**Appendix: supplementary figures**


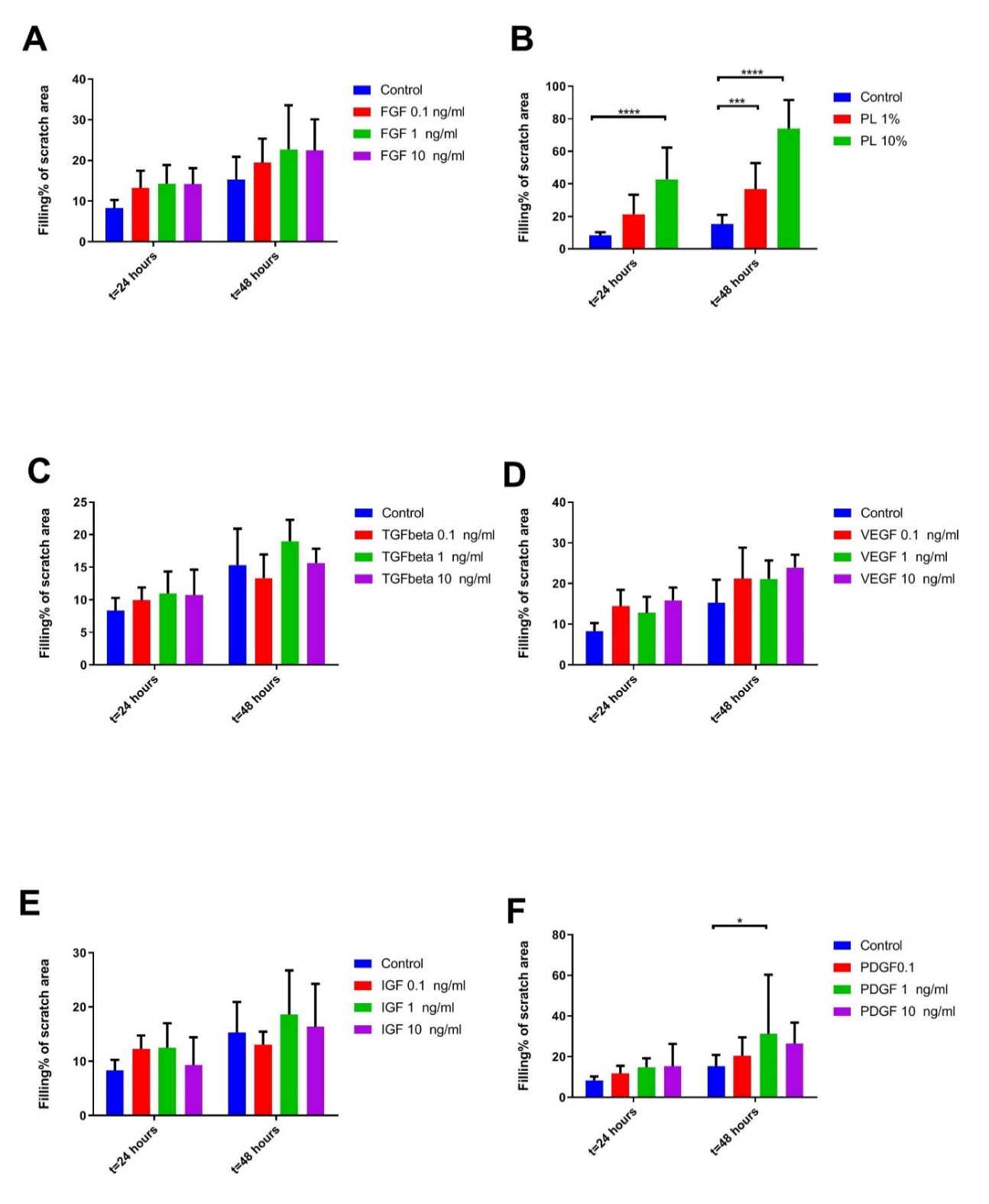


**Supplementary figure 1**: Migration of meniscus cells in the microwound assay with (A) fibroblast growth factor (FGF), (B) platelet lysate (PL), (C) transforming growth factor beta 1 (TGF-β1), (D) vascular endothelial growth factor (VEGF), (E) insulin-like growth factor-1 (IGF-1), and (F) platelet-derived growth factor (PDGF). *, p<0.05; *** p<0.001; ****, p<0.0001


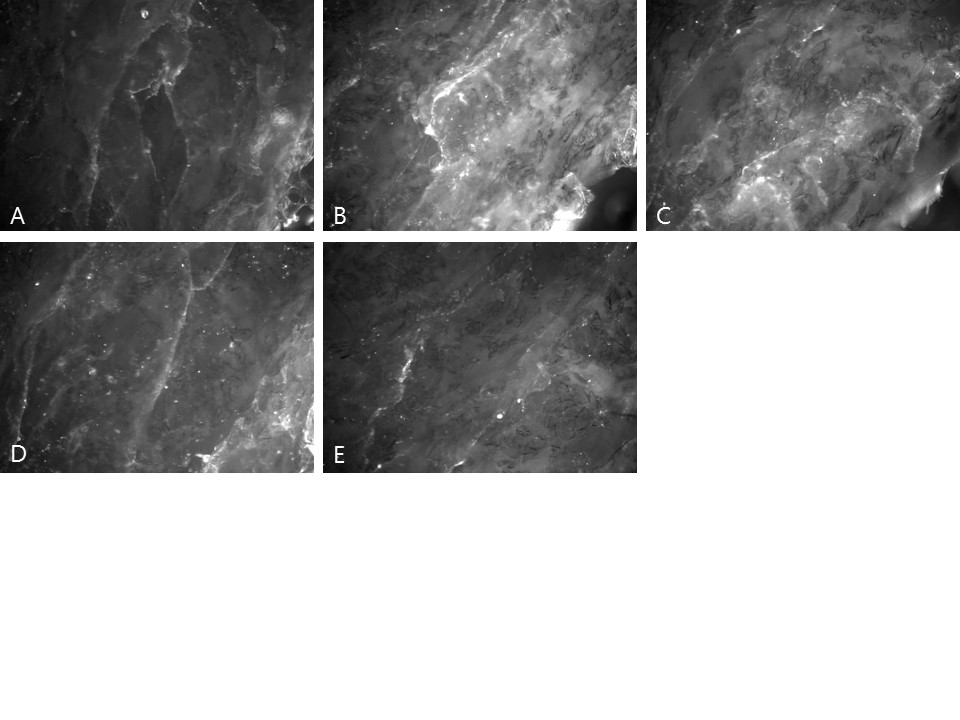
**Supplementary figure 2:** Immobilization of vascular endothelial growth factor (VEGF) on functionalized Collagen Meniscus Implants (CMI®) visualized using a fluorescently labeled anti-VEGF antibody. Image A-E show example images at different locations in the CMI® with EDC/NHS, VEGF binding peptide and VEGF.


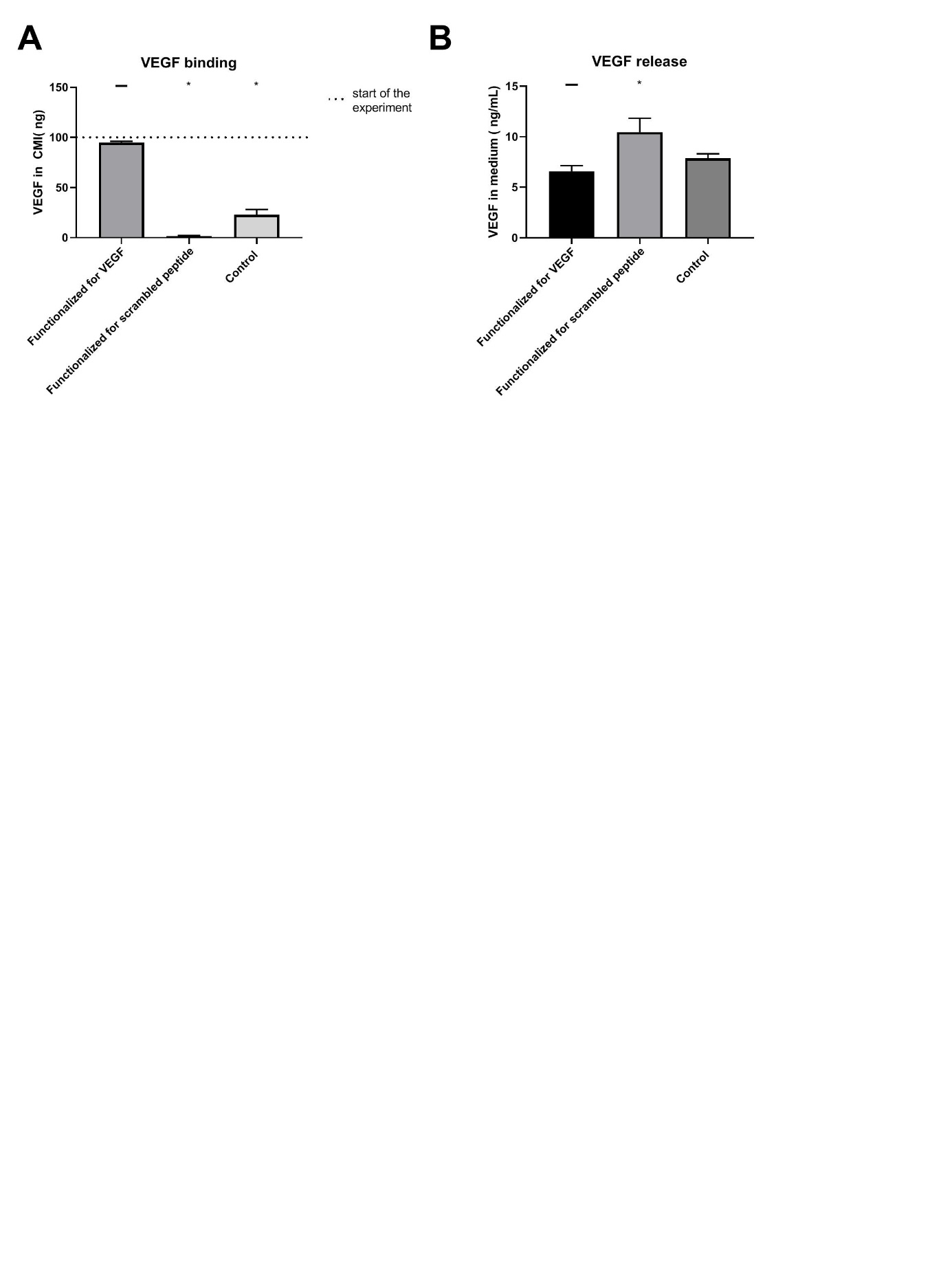


**Supplementary figure 3**: Vascular Endothelial Growth Factor (VEGF) binding in the collagen meniscus implant (CMI®), after incubating the CMI® with 100ng VEGF (A). VEGF released into the medium in 7 days culture (B).
